# Chemical principles underpinning the performance of the metal–organic framework HKUST-1

**DOI:** 10.1039/c5sc01489a

**Published:** 2015-05-11

**Authors:** Christopher H. Hendon, Aron Walsh

**Affiliations:** a Department of Chemistry , University of Bath , Claverton Down , Bath , BA2 7AY , UK . Email: a.walsh@bath.ac.uk ; Tel: +44 (0)1225 384913

## Abstract

A common feature of multi-functional metal–organic frameworks is a metal dimer in the form of a paddlewheel, as found in the structure of Cu_3_(**btc**)_2_ (HKUST-1). The HKUST-1 framework demonstrates exceptional gas storage, sensing and separation, catalytic activity and, in recent studies, unprecedented ionic and electrical conductivity. These results are a promising step towards the real-world application of metal–organic materials. In this perspective, we discuss progress in the understanding of the electronic, magnetic and physical properties of HKUST-1, representative of the larger family of Cu···Cu containing metal–organic frameworks. We highlight the chemical interactions that give rise to its favourable properties, and which make this material well suited to a range of technological applications. From this analysis, we postulate key design principles for tailoring novel high-performance hybrid frameworks.

## Abstract 2

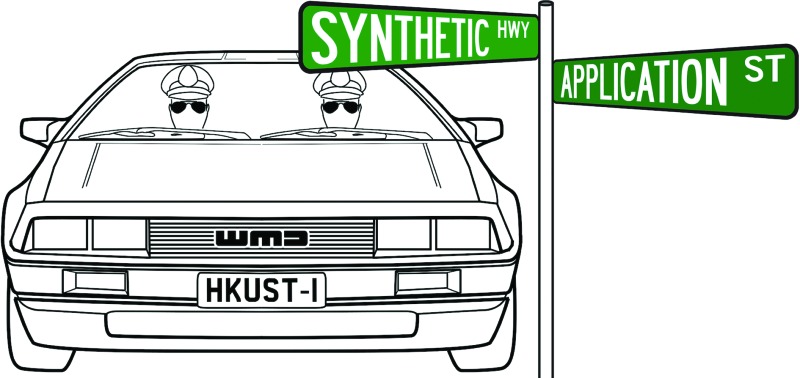
HKUST-1 has emerged as the bastion of multifunctional hybrid solids; we discuss the past, present and future of Cu-based metal–organic frameworks.

## 1 Introduction

The structural diversity of metal–organic frameworks (MOFs)^1^ has stimulated research efforts in the fields of gas storage and sensing,^2^,^3^ catalysis,^4^,^5^ and more recently, as electroactive materials in devices.^6^–^10^ Many studies have focused on the syntheses of ‘ultra-high porosity’ materials^11^,^12^ with the prospect of tailor-made gas vessels for selective uptake and storage.^13^–^18^ Whilst it is recognised that these organic–inorganic compounds can exhibit an extensive range of properties relevant to solid-state chemistry and physics – including magnetism, ferroelectricity and electrochromism – electronic structure modulation has been a secondary consideration in hybrid materials design.^19^

**Fig. 1:**
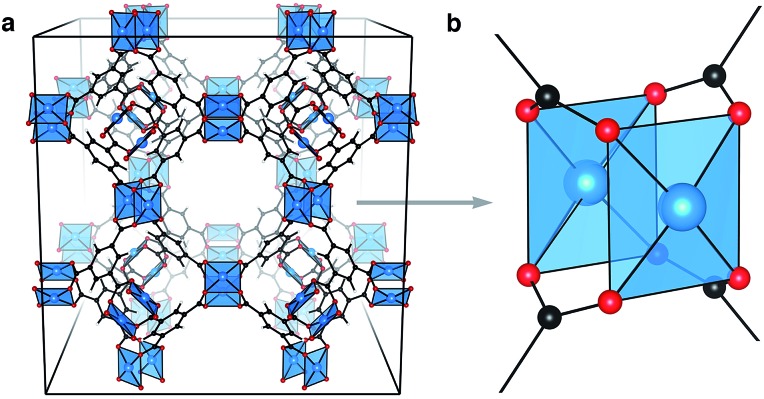
Cu_3_(**btc**)_2_ (HKUST-1) crystallises in the cubic space group *Fm*3*m*. Shown is the coordinatively unsaturated (anhydrous) structure, (a). Each crystallographic unit cell features 32 Cu···Cu paddlewheel motifs, (b), linked by the aromatic tricarboxylate, **btc**, 1,3,5-benzene tricarboxylate.

From the plethora of reported MOFs, some of the highest performing multi-application materials feature an array of Cu···Cu paddlewheel secondary building units (SBUs), shown truncated in Fig. 1b. There are many examples of organic–inorganic frameworks featuring this motif (*e.g.* HKUST-1,^20^ NU-111,^21^ PCN-14 (ref. 22) and NOTT-115 (ref. 23)), with varying porosity and aperture depending on the organic linker selection.^24^,^25^ Porous frameworks have been used in the storage of gas,^26^ but all have shown significant retention and selectivity of gaseous mixtures.^27^–^29^ It is not clear that the high porosity of these materials, attributed to the large organic linkers, correlates with high performance. Indeed, HKUST-1 is one of the more dense porous MOFs.

HKUST-1, (Cu_3_(**btc**)_2_, Fig. 1a), is a framework composed of an array of 32 Cu···Cu paddlewheels per crystallographic unit cell, connected in three dimensions by 1,3,5-benzene tricarboxylate (**btc**). Like cupric acetate, this ligand arrangement results in two coordinatively unsaturated Cu sites per paddlewheel which polar molecules can interact with. The paddlewheels are stable in both the coordinatively unsaturated and saturated arrangements.^30^ In the original preparation of HKUST-1, Cu_3_(**btc**)_2_ crystallises with stoichiometric amounts of water coordinated to each Cu^2+^ ion. Anhydrous (or activated) HKUST-1 is realised by gentle heating under low pressure, resulting in the chemically activated structure with exposed Cu^2+^ sites.

MOFs can be complex in both chemistry and geometry. In an attempt to simplify discussions of these frameworks several shorthand naming systems have been devised. The most common is an arbitrary alphanumeric string, often indicating the institution where the material was first isolated (*e.g.* HKUST-1 as a description of Cu_3_(**btc**)_2_). An alternative nomenclature describes the structural topology.^31^ For example, Cu_3_(**btc**)_2_ crystallises with so-called *tbo* topology in space group *Fm*3*m*, which relates to ‘twisted’ boracite (the mineral form of calcium borate). There are many Cu-based MOFs that deviate from the *tbo* type. For example, Eddaoudi and co-workers reported a Cu-containing MOF of type *rht*,^32^ and there are further reports including *nbo*, *cuo*, *ftw*, *eto*, *gea*, *rht* and *agw* topologies.^33^–^36^ The electronic structure and applications described herein relate specifically to HKUST-1, but the principles should be transferable to any of the Cu···Cu containing MOFs.

Activated HKUST-1 has demonstrated remarkable gas separation and uptake. It has been shown to be both an ionic and electrical conductor, and an efficient heterogeneous catalyst, despite its pedestrian chemical composition. Both **btc** and Cu···Cu paddlewheels are not unique to HKUST-1. Indeed, **btc** is a familiar ligand in MOF chemistry.^37^,^38^ Thus, the properties of HKUST-1 that underpin its multifunctionality stem from the combination of metal and ligand, in this specific topology. In this perspective, we explore the chemical and electronic characteristics of HKUST-1 and use them to form guidelines for future application-specific hybrid materials design.

## 2 Electronic and magnetic structure

In 1989, an extensive description of the electronic structure and optical response of cupric acetate was presented by Solomon and co-workers.^39^ Solomon also described the electronic structure of these cupric oxide paddle wheels in relation to biological systems.^40^ Here, the Cu^2+^ ions have a d^9^ electronic configuration and are separated by 2.65 Å. This short bond-length results in a strong antiferromagnetic (AFM) interaction within the paddle-wheel (Cu^↑^···Cu^↓^), with no appreciable interaction between adjacent paddlewheels.

**Fig. 2:**
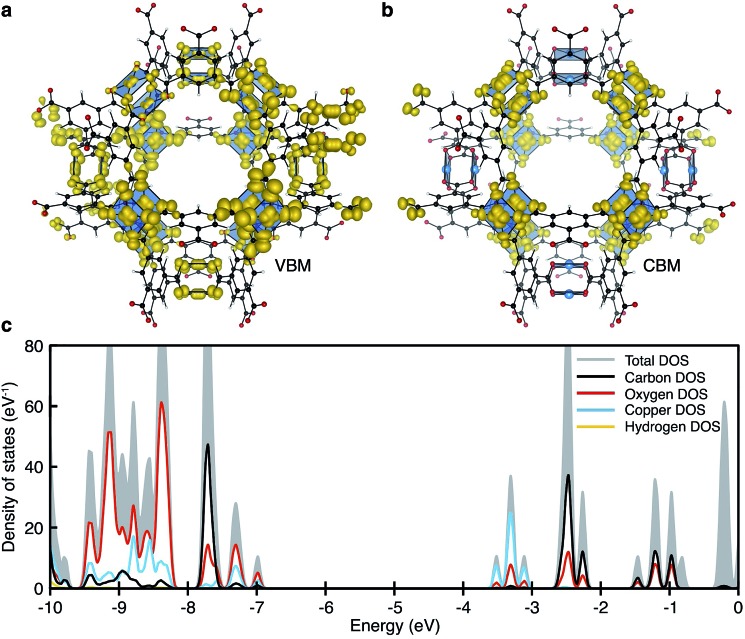
The electronic structure of HKUST-1 as computed from hybrid density functional theory (full details in ref. 41). The valence band maximum (a) and conduction band minimum (b) electronic contributions are localised on the cupric acetate motifs, with minimal influence of the aromatic groups. The electronic density of states (DOS) (c) for the ground-state antiferromagnetic configuration is characteristic of a wide band gap insulator.

**Fig. 3:**
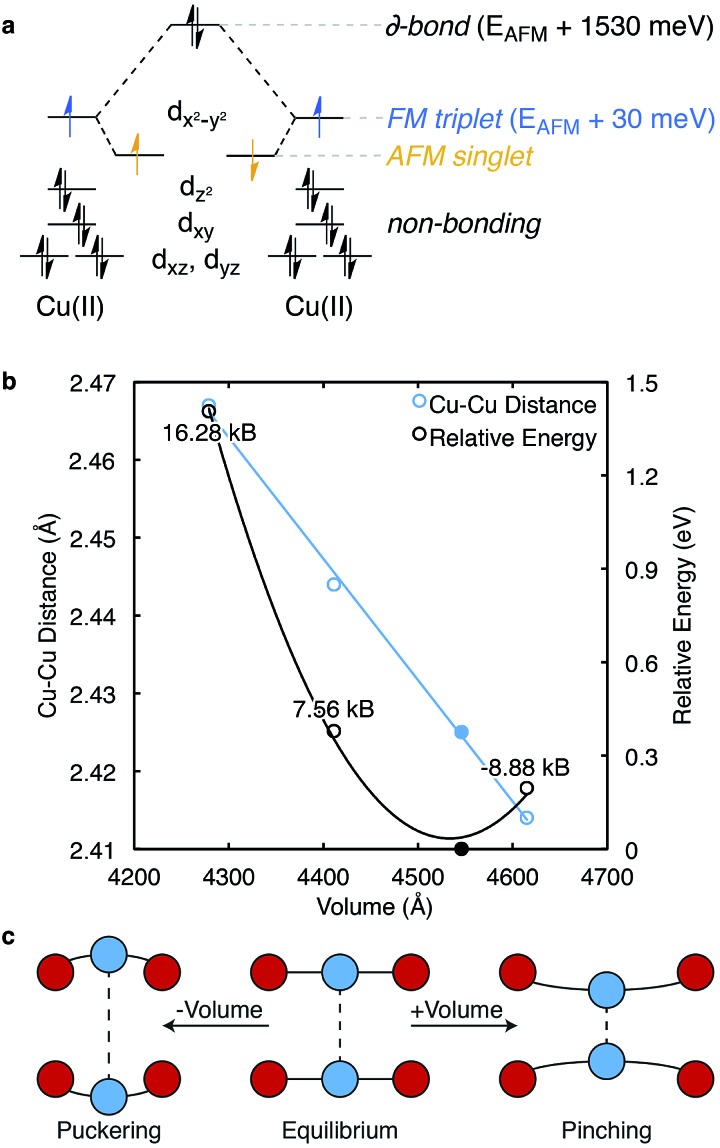
Each Cu···Cu paddlewheel has several possible spin states, some of which are summarised in the crystal field splitting diagram, (a). The ground-state of anhydrous HKUST-1 is an antiferromagnetically (AFM) coupled spin singlet, with the ferromagnetic (FM) triplet thermally accessible at room temperature. A distinct Cu···Cu bond is realised through a (closed-shell singlet) δ-bond and is 1.53 eV per Cu···Cu motif less stable than the AFM state. The Cu···Cu distance is inversely proportional to cell volume. Deviations from the equilibrium structure (the structure obtained from the black point in (b)) causes pinching or puckering of the Cu···Cu, (c), upon compression or dilation, respectively.

One decade later, the synthesis and characterisation of HKUST-1 was achieved by Williams and co-workers.^20^ The local environment of Cu^2+^ is similar to cupric acetate, with the valence and conduction bands centred on cupric acetate-like orbitals (Fig. 2). A range of spin configurations are again possible for Cu···Cu, summarised in Fig. 3a. These can be isolated from first-principles electronic structure calculations and placed in order of increasing stability: a δ-bond state (a closed-shell singlet), a ferromagnetic (FM) state (an open-shell triplet), and an antiferromagnetic state (an open-shell singlet).^41^

Following cupric acetate, HKUST-1 (both hydrated and activated) exhibits an AFM ground state, while the FM and δ-bond states may be populated by temperature and/or photo-excitation. Williams and co-workers demonstrated this effect, showing an increase of the magnetic susceptibility at temperatures greater than 100 K.^42^ In a recent study by Pöppl and co-workers, the AFM ground-state was confirmed with a range of electron spin resonance techniques, with evidence for an *S* = 1 excited spin state.^43^

From first-principles calculations, the AFM state is 1.53 eV (148 kJ mol^–1^) per paddlewheel more stable than the δ-bond state, and 0.03 eV (3 kJ mol^–1^) per paddlewheel more stable than the FM state, Fig. 3a. From the negative exchange interaction between nearest-neighbour Cu ions, a Néel temperature of 348 K was predicted.^44^ Treatment of the correct magnetic structure is critical in calculations of the electronic structure of HKUST-1 (and related open-shell frameworks). A closed-shell (spin restricted) calculation forces the δ-bond formation, which is one of many low energy excited electronic states.

There is evidence for magnetoelastic coupling in this system. The spin state of the Cu ions is sensitive to the lattice volume (pressure) and spin flipping is observed both experimentally and computationally upon increases (*ca.* 0.1 Å) in the Cu···Cu distance. Surprisingly, HKUST-1 shows a decrease (pinching) in Cu···Cu distance with an increase in cell volume, as shown in blue, Fig. 3b. A crystal subject to hydrostatic pressure will result in a greater Cu···Cu separation, or puckering. An estimate of these pressures is shown in Fig. 3.

Under high external pressure, the electronic structure is altered to a non-zero spin configuration. As the Cu···Cu distance is increased, the FM arrangement is favoured, with each paddlewheel converting to a spin triplet. Under expansion (*e.g.* at higher *T*) the metals are brought together, which subsequently destabilises both the occupied d_*z*^2^_ and partially occupied d_*x*^2^–*y*^2^_ orbitals, but the AFM state remains. Changes in effective mechanical pressure can also be induced by low loading levels of gaseous absorbates, which puckers the Cu···Cu motif, Fig. 3c.^45^–^47^

Complex magnetic behaviour was observed by the inclusion of a di-*tert*-butyl nitroxide radical, which both coordinated and coupled to the Cu ions.^48^ It is not surprising HKUST-1 interacts strongly with free radicals. As we will discuss in the catalysis section, this experiment provides a promising avenue for radical heterogeneous catalysis. The coordination process increases electron density around the Cu^2+^ d_*z*^2^_ orbitals: the destabilisation does not alter the AFM arrangement, but rather causes a gradual crystallographic expansion due to the increase in d_*z*^2^_ repulsion. The magnetic configuration not only affects the local Cu···Cu electronic structure, but also the solid-state workfunction.

**Fig. 4:**
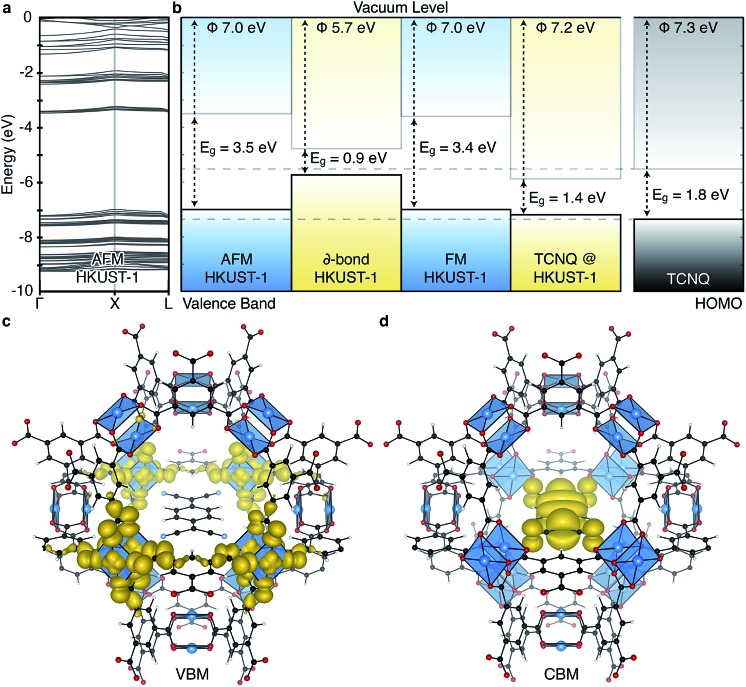
The electronic band structure of HKUST-1 in the antiferromagnetic (AFM) configuration, a, shows typical behaviour of a metal–organic framework: bands with low dispersion in reciprocal space indicating highly localised states. The three spin states per Cu···Cu paddlewheel considered result in three distinct ionisation potentials (workfunctions), shown relative to the vacuum level in (b). Inclusion of the TCNQ electron acceptor in the pores of HKUST-1 provides an electron acceptor and transport medium. Solid-state calculations of the TCNQ/HKUST-1 interface show the upper valence band of the framework is not affected by the inclusion of TCNQ, (c), however the molecular LUMO occupies a mid-gap state, (d). All calculations are based on a density functional theory with a hybrid exchange–correlation functional (HSE06).

Experimentally, workfunctions (or ionisation potentials) of MOFs are challenging to measure. MOFs are commonly unstable in polar solvents (posing problems for cyclic voltammetry measurements) and despite recent attempts at understanding surface terminations in MOFs,^49^–^51^ the exact topology is unknown (posing problems for photoemission characterisation). There are however four recent reports detailing an effective workfunction for HKUST-1,^52^–^55^ all of which suggest variable ionisation potentials depending on the spin state. Using a recent method for recovering the absolute electron energies of porous crystals,^41^ the predicted workfunctions of the various spin states of HKUST-1 may be aligned relative to vacuum, Fig. 4b. We compute the workfunctions of the AFM, FM and δ-bond state to be 7.0, 7.0 and 5.7 eV, which is in close agreement with ionisation potential measurements performed by Lee and co-workers (5.43 eV).^54^ The characteristic aquamarine hue, closely associated with the Cu···Cu motif, does not correlate with the electronic band gap of any of the spin states, it is rather attributed to optical transitions around 1.3 eV which can be associated with ligand field transitions and ligand-to-metal charge transfer.

## 3 Ionic and electrical conductivity

One can envisage many methods of activating ion conductivity in hybrid frameworks. In the case of hydrated HKUST-1, exposure to a strong base – ammonia is one example – weakens the aqueous O–H bond, resulting in labile protons.^56^,^57^ This phenomenon was further demonstrated by Hupp and co-workers, who showed an increase in proton conductivity (Grotthus mechanism^58^) upon loading of methanol in the hydrated structure.^59^

As outlined in a recent review by Shimizu and co-workers, there are several drawbacks with using MOFs as ionic conductors.^60^ Perhaps surprisingly, most reports of Cu-MOFs show stability in ammonia; the same in not true with respect to oxygen centred nucleophiles.^61^ The primarily concern is that proton hopping sites are inherently nucleophilic. A destabilisation of the nucleophilic-bound proton is required to increase the protonic conductivity; a chemical characteristic which is essentially a measurement of guest molecule acidity. Thus the increase in nucleophilicity generally results in macroscopic framework decomposition.^62^ Tominaka and Cheetham presented an alternative mode of ion conductivity in microcrystalline samples. The ionic conductivity of pressed-pelleted frameworks was observed to be extrinsic (*i.e.* the conduction occurred at the poly-crystalline grain boundaries, rather than through the proposed Grotthus mechanism that dominates single crystal samples).^63^ Besides H^+^ conductivity, a recent screening of MOFs for use as cathodes in Li–air batteries showed crystalline HKUST-1 has a particularly large discharge capacity of 4170 mA h g^–1^, in which both ion transport and storage capabilities are exploited.^64^

The ability to tune the electronic structure of MOFs, through modification of the inorganic and organic components, make them exciting candidates for electronic applications. Notably, Dincă and co-workers have shown several examples of conductive MOFs.^65^–^68^ Most metal–organic frameworks are wide band gap insulators with high resistivity.^6^,^69^,^70^ Their porosity results in electronic bands composed of highly localised orbitals, and an absence of band dispersion in reciprocal space, as shown for HKUST-1 in Fig. 4a. This suggests that the most likely mode of electronic conduction is through electron hopping, rather than band transport.^71^–^73^ In the first report of a solar cell containing HKUST-1 electron injection to TiO_2_ and hole transport to a redox electrolyte was achieved.^54^ Elsewhere, hybrid organic–inorganic perovskite structured materials have achieved notable recent success in photovoltaic applications,^74^–^76^ originating in part from the interplay between the organic and inorganic components.^77^–^80^ One distinction of the hybrid perovskites is that the extended inorganic network provides robust band, rather than hopping, conductivity.

Conventional semiconductors may be chemically doped to introduce an excess of electrons (donor states) or holes (acceptor states). The defect chemistry of MOFs is complex – many are kinetic products rather than in thermodynamic equilibrium – and there is no general approach to increase conductivity.^81^–^83^ One promising method to increase conductivity could be through increasing the MOF density. Assuming a fixed topology, this may be achieved through two approaches: (i) pre-synthetic ligand choice^84^ or (ii) loading of the MOF with electron hopping sites.^85^ In the latter approach, under-coordinated metal sites can be harnessed to transiently coordinate redox-active molecules. This principle was the basis of the seminal work of Allendorf and co-workers who demonstrated substantial electrical conductivity increase upon loading of HKUST-1 with tetracyanoquinodimethane (TCNQ).^55^

The increase in conductivity of TCNQ@HKUST-1 can be understood from basic principles of semiconductor physics.^86^ TCNQ is widely used as an electron acceptor in organic electronics. The electronic workfunction of HKUST-1 (AFM configuration) is calculated to be 7.0 eV with a band gap of 3.5 eV, Fig. 4b. Single-molecule TCNQ is predicted to have an ionisation potential of 7.3 eV with an electronic gap of 1.8 eV (at the same level of theory). Solid-state calculations of the mixed system confirm that the addition of TCNQ to coordinatively-unsaturated Cu in HKUST-1 sites provides electronic stabilisation to the framework, increasing the work function to 7.2 eV. This stabilisation originates from back-bonding capability of TCNQ which subsequently lowers the valence band maximum (VBM, dominated by cupric oxide, Fig. 4c). The lowest unoccupied molecular orbital (LUMO) of TCNQ becomes a mid gap state, consistent with its behaviour as an electron acceptor, Fig. 4d.

The inclusion of TCNQ provides a sub-framework connection – by the formation of a charge transfer salt – between the Cu paddlewheels. A similar principle of internal cross-linking modifications was demonstrated by Cohen and co-workers,^87^ but little attention has been paid to the electronic structure of the linking motifs. There is certainly scope for exploring both the inclusion of other molecules into the pores of HKUST-1 that form similar electrical contacts, with varying donor and acceptor levels.

## 4 Heterogeneous catalysis

Many hybrid frameworks have shown success as heterogeneous catalysts, in part owing to the large internal surface areas. There are reports ranging from polymerisations^88^,^89^ to C–H bond activation^90^ and CO_2_ reduction.^91^ These catalytic frameworks generally contain motifs analogous to traditional molecular catalysts, featuring the same compositional diversity, with the advantage of regular ordering and potential ease of catalyst recovery.

**Fig. 5:**
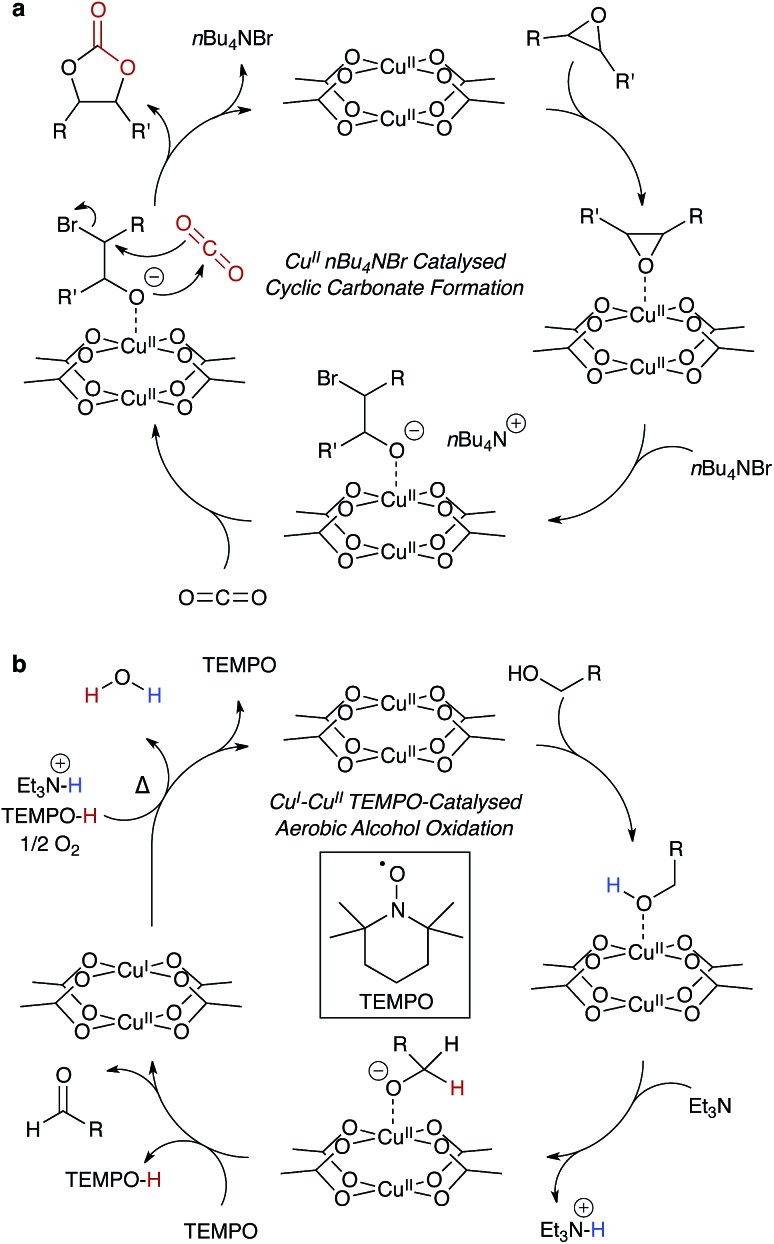
An example of a heterogeneous catalytic cycle for the formation of cyclic carbonates – cupric acetate mediated – from epoxides and CO_2_, (a). *n*Bu_4_NBr serves as both a labile nucleophile and phase transfer reagent. The same cupric acetate motif may be used as a heterogeneous mixed redox Cu^I^/Cu^II^ catalyst, (b), oxidising alcohols to aldehydes in the presence of TEMPO.

Many organic transformations do not require a redox active catalyst, but rather a combination of electron rich and deficient reagents, and an electrophilic centre, such as Cu^2+^. In principle, this type of catalysis would be ill-suited to MOFs because strongly nucleophilic reagents may result in framework decomposition. However, in reactions that are both gaseous and chemically moderate – such as the epoxide ring opening^92^,^93^ reaction shown in Fig. 5a – the porosity of MOFs comes as a benefit, with the presence of a regular array of metal centres to facilitate the stabilisation of reaction intermediates.

Ma and co-workers showed highly efficient CO_2_ conversion in HKUST-1, and quantitative catalytic turnover for their modified framework featuring an array of Cu^2+^ paddlewheels and single Cu^2+^ atoms.^91^ In their report (summarised in Fig. 5a), the phase transfer catalyst, *n*-tetrabutylammonium bromide, does not degrade the framework, and the reagents are the epoxide and CO_2_. In this case, Cu^2+^ coordinates the epoxide, weakening the C–O bond through the same mechanism that promotes proton conduction in the hydrated structure. This unconventional type of catalysis is exciting, and there is scope for chiral modifications of the framework and hence potential for asymmetric catalysis within designer porous materials.

Mixed-metal redox catalysis has also been demonstrated.^94^,^95^ In a recent report by Fischer and co-workers, the isostructural Ru-HKUST-1 was constructed with mixed Ru^II/III^ sites, which exhibited exceptional olefin hydrogenation turnover.^96^ The same Ru-HKUST-1 was reported in a study by Wade and Dincă, which also included several other interesting isostructural M-HKUST-1 analogues (M = Cr, Fe, Ni, Mo), all of which have multiple accessible oxidation states.^97^ The same principles of mixed-metal redox states can be applied to Cu-HKUST-1, forming reactive Cu^I/II^ paddlewheels. The radical stability of HKUST-1, as first demonstrated by Pöppl and co-workers,^48^ was recently revisited by Volkmer and co-workers, with the Cu···Cu paddlewheels showing mixed redox catalytic properties.^98^

Copper catalysis is important because it has cross-disciplinary relevance to both organic catalysis and biological systems. Many of these processes are involved in radical stabilisation,^99^ and this poses interesting challenges for heterogeneous development of HKUST-1 and derivatives for more complex reactions. One illuminating example is an extension to the work by Stahl and co-workers: the aerobic copper mediated radical oxidation of alcohols to aldehydes, an example is shown in Fig. 5b.^100^,^101^ This case has similar function to alcohol dehydrogenase with the inclusion of the radical initiator, TEMPO, which reduces the paddlewheel to a mixed oxidation state dimer. One possible limitation is the water by-product, so the reaction would have to be performed in conditions suitable for the liberation and reactivation of HKUST-1.

**Fig. 6:**
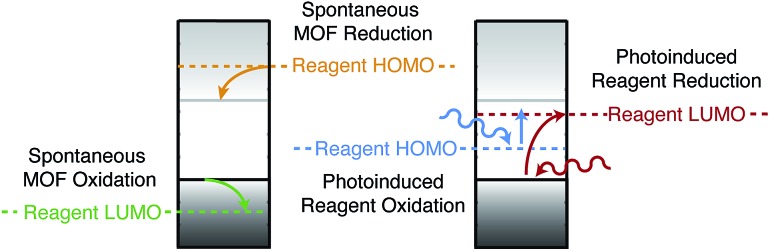
Examples of redox processes determined by the band energies of metal–organic framework and the molecular electron addition and removal energies of the reagents. A general scheme for computing the former quantities has been reported in ref. 41.

Screening porous frameworks for catalytic redox activity may be achieved with knowledge of only the energy of the bound electrons:^102^ the workfunctions from Fig. 4b. The frontier bands of HKUST-1 are cupric oxide centred, but the long-range electrostatic potential can result in substantial shifts with respect to corresponding molecular values, *i.e.* a cluster calculation of the cupric oxide paddlewheel may not reflect the electron energies of the periodic solid. In Fig. 6 we present a simple representation of four possible electronic processes that a reagent may have with the framework. If the reagent has unoccupied states lower in energy than the frameworks occupied states, there may spontaneous electron transfer from the valence band to the reagent LUMO (shown in green); this process will be limited by the instability of Cu^III^. A more probably case is the reduction of HKUST-1, if the reagent has filled states with low binding energy (shown in tangerine). The two other cases are where the guest molecule has mid-gap states that are empty (shown in red) or filled (shown in blue). The former is a simplification of the TCNQ example described in Fig. 4, whilst the latter relies on the unoccupied states of the framework.

Whilst the redox activity is linked to the absolute electron energies of the framework, these are not necessarily constant and can be influenced by changes in structure. Band modulation may prove to be an important tool for tailored catalysis. This modulation could be achieved physically by control of pressure, temperature or lattice strain.^103^ An alternative chemical route could be through modification of the organic ligand, thereby changing the structural topology or the electronic stability by electron donation or withdrawal.^104^ Two notable examples of this are the monoamination of the **btc** linker, first reported by Fröba and co-workers,^105^ and, separately, the aromatic ring catenation, first reported by Yaghi and co-workers.^106^ The latter case warranted a new name, MOF-14, as the pores were so large that interpenetration was observed. The non-interpenetrated structure should be accessible through solvent selection.

## 5 Gas storage, sensing and separation

Gaseous molecules have the potential to interact strongly with open metal sites, as defined by the electrostatic interaction between framework and absorbate. A stronger interaction is found for more polar and polarisable absorbates.^107^,^108^

HKUST-1 has the highest density of Cu···Cu motifs of any known MOF. The exposed coordination sites provide a high metal-to-unit cell ratio, making HKUST-1 an excellent material for gas uptake, sensing, and separation.^109^ However, the space inside the pore (open pore volume space) is inactive. This factor is why no MOF currently achieves the physical requirements to be a useful alternative to compressed gas storage.^110^ Thus, we focus on the potential for gas sensing and separations achieved with HKUST-1.

MOFs can be used to sense molecules in many ways. Besides the visible colour shift observed for HKUST-1 (which is even notable in the dehydration process),^55^ optical and structural techniques can be used to detect absorbate induced changes in the framework.^28^ Most MOFs contract upon low loading of guest molecules.^111^ This contraction may be observable in optical spectroscopy, but also clearly shown with X-ray diffraction.^45^ The contraction is absorbate specific, and with the knowledge of band positions changing relative to pressure, one can envisage an electrical device based on change in resistance upon change in pressure. Also, if the change in pressure is substantial, one could observe a change the spin state: magnetoelastic sensing.

Generally, the more polar the guest molecules, the stronger the interaction with the framework. This fundamental physical concept reconciles the multitude of literature showing, for instance, selective retention of CO_2_ over CH_4_: CO_2_ has a much larger quadrupole moment than CH_4_.^112^ The *single* report that challenges this principle show retention of the less polarisable *p*-xylene over *o*/*m*-xylene, but in this case it was determined that the retention was attributed to *o*- and *m*-xylene accessing otherwise inaccessible pores.^113^

HKUST-1 features three distinct internal pores, two of comparable size (aperture = 14 Å) and a smaller pore (aperture = 10 Å). Of the two larger pores, one has Cu···Cu directed into the pores (shown coordinating TCNQ in Fig. 4c), making this ideal for gas interactions. The smallest pore could be used to retain smaller molecules. Increasing the density of both open-metal site, and the framework itself, are methods of increasing the selective retention and initial gas uptake volumes, but this comes with the cost of a reduced pore aperture.

## 6 Conclusions and outlook

Considering the body of research built up over the past 15 years, it is evident that HKUST-1 is a unique metal–organic framework. The electronic characteristics that underpin the performance of the material originate from the synergy of the structural organic linker, **btc**, and the magnetically coupled Cu···Cu paddlewheel motif. Drawing from the current state of understanding – and informed by first-principles materials modelling – we can infer a set of design principles to be used as a roadmap to other high-performance applications of HKUST-1, and importantly, the design of novel functional frameworks.

### Magnetic paddlewheels

The electronic structure of the cupric acetate-derived paddlewheel motif is complex and open to changes in chemical coordination and spin. Considering magnetic modulation, the design of organic linkers that permit superexchange interactions between adjacent paddlewheels is of particular interest. The primary challenge is to modify the organic motifs such that desirable orbital symmetry is obtained, without dramatic alteration to the overall MOF structure. One potential class of ligands could be the extended linear polyalkynes, which satisfy the preliminary criteria.^114^ Alternatively, the control of spin arrangements provides scope for three-dimensional arrays of quantum data storage, although chemical modification of the framework may be necessary to prolong the spin lifetimes.^115^ Furthermore, the observation of spin flipping may eventuate in magnetoelastic chemical sensing.

### Conductive paddlewheels

Currently, HKUST-1 features the most dense array of Cu···Cu motifs of any framework, with the smallest distance between adjacent Cu···Cu motifs. It should be possible to further reducing the porosity with ligand design, whereby modulating the inter-paddlewheel distances. This could result in an increase in the orbital overlap and potentially intra-framework electron transport. The gauntlet has been thrown down to the experimentalists to realise this challenge. Increasing the size of the ligand simply decreases the Cu···Cu density. While not optimal for conductivity, such an approach can be of interest for applications such as gas storage.^116^

### ‘Rewiring’ the framework

Another method for increasing conductivity is modifications to the guest electron acceptor and donor sites. Electronic control of these guests would allow access to both the valence and conduction bands. Coordination control could provide a means to direct conductivity along specific crystallographic directions. Currently only TCNQ has been shown to increase conductivity in HKUST-1. This is not because it is unique, but rather because of its dimensions and that the electron energies coincide with HKUST-1. The same principles may be extended to any pair of materials.

### Porous catalysis

There are many opportunities for novel catalysis inside MOFs. In the case of HKUST-1, there is the potential for organic synthesis that is bio-relevant. The well-defined oxidation and spin states of the Cu···Cu motifs is desirable and there is scope for chiral catalysis in modified HKUST-1 analogues. Any material with open-metal sites can potentially be used to coordinate electron-rich motifs, thus paving way for heterogeneous catalysis in chemically robust frameworks.

### Chemical capture

Under-coordinated metal sites are ideal for increasing the local electric fields within a MOF pore. The Cu···Cu motif is particularly well-suited. The same forces that attract water and result in decomposition can be used to capture highly polar molecules like those found in nerve agents and chemical weapons. Thus, the application of MOFs for capture of polar molecules is an important variation on the usual approach that is storage of non-polar species like methane.^117^

## References

[cit1] Cheetham A. K., Rao C. N. R. (2007). Science.

[cit2] Brown J. W., Henderson B. L., Kiesz M. D., Whalley A. C., Morris W., Grunder S., Deng H., Furukawa H., Zink J. I., Stoddart J. F., Yaghi O. M. (2013). Chem. Sci..

[cit3] Mason J. A., Veenstra M., Long J. R. (2014). Chem. Sci..

[cit4] Llabrés i Xamena F., Casanova O., Galiassotailleur R., Garcia H., Corma A. (2008). J. Catal..

[cit5] Dhakshinamoorthy A., Alvaro M., Garcia H. (2012). Chem. Commun..

[cit6] Stavila V., Talin A. A., Allendorf M. D. (2014). Chem. Soc. Rev..

[cit7] Jin S., Son H.-J., Farha O. K., Wiederrecht G. P., Hupp J. T. (2013). J. Am. Chem. Soc..

[cit8] Hendon C. H., Tiana D., Walsh A. (2012). Phys. Chem. Chem. Phys..

[cit9] Hendon C. H., Tiana D., Vaid T. P., Walsh A. (2013). J. Mater. Chem. C.

[cit10] Tiana D., Hendon C. H., Walsh A., Vaid T. P. (2014). Phys. Chem. Chem. Phys..

[cit11] Banerjee R., Phan A., Wang B., Knobler C., Furukawa H., O'Keeffe M., Yaghi O. M. (2008). Science.

[cit12] Furukawa H., Cordova K. E., O'Keeffe M., Yaghi O. M. (2013). Science.

[cit13] Dzubak A., Lin L., Kim J., Swisher J., Poloni R., Maximoff S. N., Smit B., Gagliardi L. (2012). Nat. Chem..

[cit14] Lee K., Isley W. C., Dzubak A. L., Verma P., Stoneburner S. J., Lin L.-C., Howe J. D., Bloch E. D., Reed D. A., Hudson M. R., Brown C. M., Long J. R., Neaton J. B., Smit B., Cramer C. J., Truhlar D. G., Gagliardi L. (2014). J. Am. Chem. Soc..

[cit15] Lin X., Jia J., Zhao X., Thomas K. M., Blake A. J., Walker G. S., Champness N. R., Hubberstey P., Schröder M. (2006). Angew. Chem., Int. Ed..

[cit16] Chavan S., Vitillo J. G., Gianolio D., Zavorotynska O., Civalleri B., Jakobsen S., Nilsen M. H., Valenzano L., Lamberti C., Lillerud K. P., Bordiga S. (2012). Phys. Chem. Chem. Phys..

[cit17] Kim H., Park J., Jung Y. (2013). Phys. Chem. Chem. Phys..

[cit18] Lucena S., Mileo P., Silvino P., Cavalcante Jr C. (2011). J. Am. Chem. Soc..

[cit19] Kieslich G., Sun S., Cheetham T. (2014). Chem. Sci..

[cit20] Chui S. S., Lo S. M., Charmant J. P. H., Orpen A. G., Williams I. D. (1999). Science.

[cit21] Peng Y., Srinivas G., Wilmer C. E., Eryazici I., Snurr R. Q., Hupp J. T., Yildirim T., Farha O. K. (2013). Chem. Commun..

[cit22] Ma S., Sun D., Simmons J. M., Collier C. D., Yuan D., Zhou H.-C. (2008). J. Am. Chem. Soc..

[cit23] Yan Y., Blake A. J., Lewis W., Barnett S. A., Dailly A., Champness N. R., Schröder M. (2011). Chem.–Eur. J..

[cit24] Farha O. K., Wilmer C. E., Eryazici I., Hauser B. G., Parilla P. A., O'Neill K., Sarjeant A. A., Nguyen S. T., Snurr R. Q., Hupp J. T. (2012). J. Am. Chem. Soc..

[cit25] Li L., Bell J. G., Tang S. F., Lv X., Wang C., Xing Y., Zhao X., Thomas K. M. (2014). Chem. Mater..

[cit26] Peng Y., Krungleviciute V., Eryazici I., Hupp J. T., Farha O. K., Yildirim T. (2013). J. Am. Chem. Soc..

[cit27] Yan Y., Yang S., Blake A. J., Schröder M. (2014). Acc. Chem. Res..

[cit28] Allendorf M. D., Houk R. J. T., Andruszkiewicz L., Talin A. A., Pikarsky J., Choudhury A., Gall K. A., Hesketh P. J. (2008). J. Am. Chem. Soc..

[cit29] Hendon C. H., Wittering K. E., Chen T.-H., Kaveevivitchai W., Popov I., Butler K. T., Wilson C. C., Cruickshank D. L., Miljanić O. Š., Walsh A. (2015). Nano Lett..

[cit30] Peterson V. K., Southon P. D., Halder G. J., Price D. J., Bevitt J. J., Kepert C. J. (2014). Chem. Mater..

[cit31] OKeeffe M., Peskov M. A., Ramsden S. J., Yaghi O. M. (2008). Acc. Chem. Res..

[cit32] Nouar F., Eubank J. F., Bousquet T., Wojtas L., Zaworotko M. J., Eddaoudi M. (2008). J. Am. Chem. Soc..

[cit33] Liu K., Li B., Li Y., Li X., Yang F., Zeng G., Peng Y., Zhang Z., Li G., Shi Z., Feng S., Song D. (2014). Chem. Commun..

[cit34] Liang Z., Du J., Sun L., Xu J., Mu Y., Li Y., Yu J., Xu R. (2013). Inorg. Chem..

[cit35] Cai J., Rao X., He Y., Yu J., Wu C., Zhou W., Yildirim T., Chen B., Qian G. (2014). Chem. Commun..

[cit36] Guillerm V., Weselinski L. J., Belmabkhout Y., Cairns A. J., D'Elia V., Wojtas L., Adil K., Eddaoudi M. (2014). Nat. Chem..

[cit37] He Y., Li B., O'Keeffe M., Chen B. (2014). Chem. Soc. Rev..

[cit38] Lu W., Wei Z., Gu Z.-Y., Liu T.-F., Park J., Park J., Tian J., Zhang M., Zhang Q., Gentle III T., Bosch M., Zhou H.-C. (2014). Chem. Soc. Rev..

[cit39] Ross P. K., Allendorf M. D., Solomon E. I. (1989). J. Am. Chem. Soc..

[cit40] Solomon E. I., Hemming B. L. and Root D. E., Electronic Structures of Active Sites in Copper Proteins: Coupled Binuclear and Trinuclear Cluster Sites, Springer, Netherlands, 1993, pp. 3–5.

[cit41] Butler K. T., Hendon C. H., Walsh A. (2014). J. Am. Chem. Soc..

[cit42] Zhang X. X., Chui S. S.-Y., Williams I. D. (2000). J. Appl. Phys..

[cit43] Pöppl A., Kunz S., Himsl D., Hartmann M. (2008). J. Phys. Chem. C.

[cit44] Tiana D., Hendon C. H., Walsh A. (2014). Chem. Commun..

[cit45] Neimark A. V., Coudert F.-X., Boutin A., Fuchs A. H. (2010). J. Phys. Chem. Lett..

[cit46] Prestipino C., Regli L., Vitillo J., Bonino F., Damin A., Lamberti C., Zecchina A., Solari P. L., Kongshaug K. O., Bordiga S. (2006). Chem. Mater..

[cit47] Brown C. M., Liu Y., Yildirim T., Peterson V. K., Kepert C. J. (2009). Nanotechnology.

[cit48] Jee B., Koch K., Moschkowitz L., Himsl D., Hartman M., Pöppl A. (2011). J. Phys. Chem. Lett..

[cit49] Zacher D., Schmid R., Wöll C., Fischer R. A. (2011). Angew. Chem., Int. Ed..

[cit50] Amirjalayer S., Tafipolsky M., Schmid R. (2014). J. Phys. Chem. Lett..

[cit51] McGuire C. V., Forgan R. S. (2015). Chem. Commun..

[cit52] Pohle R., Tawil A., Davydovskaya P., Fleischer M. (2011). Procedia Eng..

[cit53] Davydovskaya P., Pohle R., Tawil A., Fleischer M. (2013). Sens. Actuators, B.

[cit54] Lee D., Shinde D., Yoon S. (2014). J. Phys. Chem. C.

[cit55] Talin A. A., Centrone A., Ford A. C., Foster M. E., Stavila V., Haney P., Kinney R. A., Szalai V., El Gabaly F., Yoon H. P., Léonard F., Allendorf M. D. (2014). Science.

[cit56] Peterson G. W., Wagner G. W., Balboa A., Mahle J., Sewell T., Karwacki C. J. (2009). J. Phys. Chem. C.

[cit57] Jee B., Eisinger K., Gul-E-Noor F., Bertmer M., Hartmann M., Himsl D., Pöppl A. (2010). J. Phys. Chem. C.

[cit58] Agmon N. (1995). Chem. Phys. Lett..

[cit59] Jeong N. C., Samanta B., Lee C. Y., Farha O. K., Hupp J. T. (2012). J. Am. Chem. Soc..

[cit60] Ramaswamy P., Wong N. E., Shimizu G. K. H. (2014). Chem. Soc. Rev..

[cit61] Campbell M. G., Sheberla D., Liu S. F., Swager T. M., Dincă M. (2015). Angew. Chem., Int. Ed..

[cit62] Mustafa D., Breynaert E., Bajpe S. R., Martens J. A., Kirschhock C. E. A. (2011). Chem. Commun..

[cit63] Tominaka S., Cheetham A. K. (2014). RSC Adv..

[cit64] Wu D., Guo Z., Yin X., Pang Q., Tu B., Zhang L., Wang Y.-G., Li Q. (2014). Adv. Mater..

[cit65] Narayan T., Miyakai T., Seki S., Dinc M. (2012). J. Am. Chem. Soc..

[cit66] Sun L., Miyakai T., Seki S., Dincă M. (2013). J. Am. Chem. Soc..

[cit67] Sheberla D., Sun L., Blood-Forsythe M. A., Er S., Wade C. R., Brozek C. K., Aspuru-Guzik A., Dincă M. (2014). J. Am. Chem. Soc..

[cit68] Park S. S., Hontz E. R., Sun L., Hendon C. H., Walsh A., Van Voorhis T., Dincă M. (2015). J. Am. Chem. Soc..

[cit69] Allendorf M. D., Schwartzberg A., Stavila V., Talin A. A. (2011). Chem.–Eur. J..

[cit70] Falcaro P., Ricco R., Doherty C. M., Liang K., Hill A. J., Styles M. J. (2014). Chem. Soc. Rev..

[cit71] Yu P. Y. and Cardona M., Fundamentals of Semiconductors, Springer, Berlin, 3rd edn, 2005.

[cit72] Turner D. L., Vaid T. P., Stephens P. W., Stone K. H., DiPasquale A. G., Rheingold A. L. (2008). J. Am. Chem. Soc..

[cit73] Loera-Serna S., Oliver-Tolentino M. A., de Lourdes López-Núñez M., Santana-Cruz A., Guzmán-Vargas A., Cabrera-Sierra R., Beltrán H. I., Flores J. (2012). J. Alloys Compd..

[cit74] Kojima A., Teshima K., Shirai Y., Miyasaka T. (2009). J. Am. Chem. Soc..

[cit75] Im J.-H., Lee C.-R., Lee J.-W., Park S.-W., Park N.-G. (2011). Nanoscale.

[cit76] Lee M. M., Teuscher J., Miyasaka T., Murakami T. N., Snaith H. J. (2012). Science.

[cit77] Frost J. M., Butler K. T., Brivio F., Hendon C. H., Van Schilfgaarde M., Walsh A. (2014). Nano Lett..

[cit78] Frost J. M., Butler K. T., Walsh A. (2014). APL Mater..

[cit79] Hendon C. H., Yang R. X., Burton L. A., Walsh A. (2015). J. Mater. Chem. A.

[cit80] Walsh A., Scanlon D. O., Chen S., Gong X. G., Wei S.-H. (2015). Angew. Chem., Int. Ed..

[cit81] Walsh A. (2010). J. Phys. Chem. Lett..

[cit82] Walsh A. (2011). Proc. R. Soc. A.

[cit83] Cliffe M. J., Wan W., Zou X., Chater P. A., Kleppe A. K., Tucker M. G., Wilhelm H., Funnell N. P., Coudert F.-X., Goodwin A. L. (2014). Nat. Commun..

[cit84] Chen T.-H., Popov I., Kaveevivitchai W., Miljanić O. Š. (2014). Chem. Mater..

[cit85] Foster M. E., Azoulay J. D., Wong B. M., Allendorf M. D. (2014). Chem. Sci..

[cit86] Sze S. and Lee M.-K., Semiconductor Devices Physics and Technology, John Wiley & Sons, Singapore, 3rd edn, 2013.

[cit87] Allen C. A., Cohen S. M. (2014). Inorg. Chem..

[cit88] Uemura T., Kitaura R., Ohta Y., Nagaoka M., Kitagawa S. (2006). Angew. Chem., Int. Ed..

[cit89] Distefano G., Suzuki H., Tsujimoto M., Isoda S., Bracco S., Comotti A., Sozzani P., Uemura T., Kitagawa S. (2013). Nat. Chem..

[cit90] Xiao D. J., Bloch E. D., Mason J. A., Queen W. L., Hudson M. R., Planas N., Borycz J., Dzubak A. L., Verma P., Lee K., Bonino F., Crocellà V., Yano J., Bordiga S., Truhlar D. G., Gagliardi L., Brown C. M., Long J. R. (2014). Nat. Chem..

[cit91] Gao W.-Y., Chen Y., Niu Y., Williams K., Cash L., Perez P. J., Wojtas L., Cai J., Chen Y.-S., Ma S. (2014). Angew. Chem., Int. Ed..

[cit92] Johnson M. R., Nakata T., Kishi Y. (1979). Tetrahedron Lett..

[cit93] Jiang D., Mallat T., Krumeich F., Baiker A. (2008). J. Catal..

[cit94] Leong C. F., Chan B., Faust T. B., D'Alessandro D. M. (2014). Chem. Sci..

[cit95] Cozzolino A. F., Brozek C. K., Palmer R. D., Yano J., Li M., Dincă M. (2014). J. Am. Chem. Soc..

[cit96] Kozachuk O., Luz I., Llabrés I Xamena F. X., Noei H., Kauer M., Albada H. B., Bloch E. D., Marler B., Wang Y., Muhler M., Fischer R. A. (2014). Angew. Chem., Int. Ed..

[cit97] Wade C. R., Dincă M. (2012). Dalton Trans..

[cit98] Grzywa M., Gessner C., Bredenkoetter B., Denysenko D., Van Leusen J., Kögerler P., Klemm E., Volkmer D. (2014). Dalton Trans..

[cit99] Hendon C. H., Carbery D. R., Walsh A. (2014). Chem. Sci..

[cit100] Hoover J. M., Ryland B. L., Stahl S. S. (2013). ACS Catal..

[cit101] Ryland B. L., Stahl S. S. (2014). Angew. Chem., Int. Ed..

[cit102] Chambers M. B., Wang X., Elgrishi N., Hendon C. H., Walsh A., Bonnefoy J., Canivet J., Quadrelli E. A., Farrusseng D., Mellot-Draznieks C., Fontecave M. (2015). ChemSusChem.

[cit103] Butler K. T., Hendon C. H., Walsh A. (2014). ACS Appl. Mater. Interfaces.

[cit104] Hendon C. H., Tiana D., Fontecave M., Sanchez C., D'arras L., Sassoye C., Rozes L., Mellot-Draznieks C., Walsh A. (2013). J. Am. Chem. Soc..

[cit105] Peikert K., Hoffmann F., Fröba M. (2012). Chem. Commun..

[cit106] Chen B., Eddaoudi M., Hyde S. T., O'Keeffe M., Yaghi O. M. (2001). Science.

[cit107] Meek S., Teich-McGoldrick S., Perry IV J. J., Greathouse J. A., Allendorf M. D. (2012). J. Phys. Chem. C.

[cit108] Hulvey Z., Lawler K., Qiao Z., Zhou J., Fairen-Jimenez D., Snurr R. Q., Ushakov S. V., Navrotsky A., Brown C. M., Forster P. M. (2013). J. Phys. Chem. C.

[cit109] He Y., Zhou W., Qian G., Chen B. (2014). Chem. Soc. Rev..

[cit110] Lee S.-J., Bae Y.-S. (2014). J. Phys. Chem. C.

[cit111] Neimark A. V., Coudert F.-X., Triguero C., Boutin A., Fuchs A. H., Beurroies I., Denoyel R. (2011). Langmuir.

[cit112] Hickey A. L., Rowley C. N. (2014). J. Phys. Chem. A.

[cit113] Vermoortele F., Maes M., Moghadam P. Z., Lennox M. J., Ragon F., Boulhout M., Biswas S., Laurier K. G. M., Beurroies I., Denoyel R., Roeffaers M., Stock N., Dren T., Serre C., De Vos D. E. (2011). J. Am. Chem. Soc..

[cit114] Hendon C. H., Tiana D., Murray A. T., Carbery D. R., Walsh A. (2013). Chem. Sci..

[cit115] Weber J. R., Koehl W. F., Varley J. B., Janotti A., Buckley B. B., Van de Walle C. G., Awschalom D. D. (2010). Proc. Natl. Acad. Sci. U. S. A..

[cit116] Duan X., Wu C., Xiang S., Zhou W., Yildirim T., Cui Y., Yang Y., Chen B., Qian G. (2015). Inorg. Chem..

[cit117] Decoste J. B., Peterson G. W. (2014). Chem. Rev..

